# Heme Oxygenase-1 Protects Hair Cells From Gentamicin-Induced Death

**DOI:** 10.3389/fncel.2022.783346

**Published:** 2022-04-13

**Authors:** Yang Yang, Xin Chen, Keyong Tian, Chaoyong Tian, Liyang Chen, Wenjuan Mi, Qiong Li, Jianhua Qiu, Ying Lin, Dingjun Zha

**Affiliations:** ^1^Department of Otolaryngology-Head and Neck Surgery, Xijing Hospital, Air Force Military Medical University, Xi’an, China; ^2^Smartgenomics Technology Institute, Tianjin, China

**Keywords:** gentamicin, ototoxicity, differential gene expression, HO-1, hair cells, Nrf2

## Abstract

Gentamicin ototoxicity can generate free radicals within the inner ear, leading to permanent damage to sensory hair cells (HCs) and eventually hearing loss. The following study examined the alterations of oxidative damage-related genes in the cochlea and important molecules responsible for oxidation following gentamicin injury *in vitro*. The RT^2^ Profiler polymerase chain reaction (PCR) array was used to screen candidate targets for treatment to prevent hearing loss caused by gentamicin. We found that during gentamicin-induced death in HCs, Heme oxygenase-1 (HO-1) had a high fold change in the HCs of the cochlea. Moreover, the use of CoPPIX to induce HO-1 inhibited gentamicin-induced HC death, while HO-1 inhibitors ZnPPIX after CoPPIX reversed this process. Furthermore, the inhibitors of NF-E2-related factor-2 (Nrf2) reduced the expression of HO-1 and inhibited the protective effect of HO-1 after gentamicin, thus suggesting that the Nrf2/HO-1 axis might regulate gentamicin-associated ototoxicity. We further demonstrated that induction of HO-1 up-regulated the expression of Nrf2 in both cochlear and HEI-OC1 cells. In summary, these findings indicated that HO-1 protects HCs from gentamicin by up-regulating its expression in HCs and interacting with Nrf2 to inhibit reactive oxygen species (ROS).

## Introduction

Hearing loss is the most common sensory impairment worldwide. According to the WHO statistics,^1^ approximately 700 million hearing loss cases were recorded worldwide in 2018, and this number is expected to increase over 900 million by 2050 (Guo et al., 2019). Hearing loss usually results from the death of hair cells (HCs) in the inner ear. HCs constitute auditory and balance sensory cells that convert mechanical stimuli into neural signals (WenWei et al., 2018). These cells are susceptible to various stressors, such as aging, noise trauma, gene mutations, and treatment with ototoxic drugs, e.g., aminoglycosides and cisplatin (Fettiplace, 2017; Pang et al., 2019; Han et al., 2020). HC death resulting from ototoxic drugs represents an important health challenge. Aminoglycoside antibiotics remain one of the most commonly used antibiotic groups worldwide. Approximately 20% of individuals using these agents have shown serious hearing loss and/or balance damage, especially those using gentamicin (Kirst and Marinelli, 2014). Apparently, gentamicin generates free radicals within the inner ear, which leads to subsequent permanent damage to sensory cells (Zhou et al., 2018; Fujimoto and Yamasoba, 2019).

Reactive oxygen species (ROS) has a crucial role in the promotion of apoptosis by interfering mitochondrial permeability, release of cytochrome c and caspases (Pyun et al., 2011). Quan et al. (2015) demonstrated that up-regulation of Sirt3, a member of the Sirtuin family, may inhibit the production of ROS in apoptotic cells induced by gentamicin.

Moreover, preliminary experiments revealed that heme oxygenase-1 (Hmox-1/HO-1) was up-regulated in gentamicin-induced HC death. HO constitutes the rate-limiting enzymes in the process of heme degradation, causing the production of biliverdin, free iron, and CO (Medina et al., 2019). As an important protein in cell response to stress, HO-1 is triggered by numerous oxidative substances or conditions such as heme (Maines, 1988), hyperoxia (Chan Kwon et al., 2020), hypoxia (Tian et al., 2020), and electrophiles via AP-1, STAT, and Nrf2 up-regulation at the mRNA level (Alam et al., 2020; Lee et al., 2020). HO-1 is widely present in the kidneys, liver, lungs, and other organs, including the inner ear (He et al., 2019; Yi et al., 2020). Previous studies have suggested that pharmacological HO-1 activation exerts protective effect on a variety of stresses in the retina and liver following ischemia-reperfusion injury (Cheng and Rong, 2017; Hirao et al., 2020). Some reports also suggested that HO-1 has cochlear localization and is induced upon heat shock (Fairfield et al., 2004). Meanwhile, HO-1 inducers exert protective effects on cisplatin-associated HEI-OC1 cell death (Lee et al., 2019; Sun et al., 2019). Another study demonstrated that inducing HO-1 protects the organs of Corti explants from cisplatin-related HC death in newborn rats (Kim et al., 2015). The functional effect of HO-1 induction has been studied by assessing the ability of cell to resist multiple stress injuries with under- or over-expressed HO-1. These reports mainly focused on the critical cell defense effect of HO-1 on oxidative stress (Fontecha-Barriuso et al., 2020; Tian et al., 2020).

The current study focused on the alterations of oxidative damage-related genes in the cochlea following gentamicin treatment. We further aimed to quantify and characterize the differential expression of the important molecule responsible for oxidation and to clarify the protective effect of HO-1 in the process of gentamicin injury. The current study provides insight into molecular targets to prevent gentamicin ototoxicity.

## Materials and Methods

### Animals

Neonatal (P2) Sprague-Dawley (SD) rats were provided by the Laboratory Animal Center of the Air Force Medical University. All the animals were housed in an environment with a temperature of 22 ± 1°C, relative humidity of 50 ± 1%, and a light/dark cycle of 12/12 h (lights on at 8 a.m. and off at 5 p.m.). All animal studies, including the mice euthanasia procedure, were done in compliance with Air Force Medical University institutional animal care regulations and guidelines and conducted according to the AAALAC and the IACUC guidelines.

### Tissues Culture

At P2, the organs of Corti dissection from the rat’s cochlear tissue were performed. The specimens were placed in Hank’s balanced salt solution (H1045, Solabio, Beijing, China). The entire organs of Corti were cultured in Hanging Cell Culture Inserts (MCSP24H48, EMD Millipore Corp, Billerica, MA, United States). The culture medium consisted of Minimum Essential Medium supplemented with Earle’s salt and L-glutamine (11095080, Gibco, Grand Island, NY, United States), 3 mg/mL glucose, and 0.3 mg/mL penicillin (P3032, Sigma-Aldrich, St. Louis, MO, United States). The organs of Corti were cultured at 37°C in a humid environment with 5% CO_2_. The final concentrations of gentamicin (E003632-1G, Sigma-Aldrich, St. Louis, MO, United States) were 0.3 mM, 0.6 mM, and 1 mM. The HO-1 activator Co (III) protoporphyrin IX chloride (CoPPIX) (Co654, Frontier Scientific, Logan, UT, United States) was used at 5 μM in the CoPPIX group with a 12 h incubation. Tissues undergoing both CoPPIX and gentamicin treatments were first cultured with CoPPIX for 12 h, after which the medium was replaced by gentamicin-loaded medium for 24 h incubation. Meanwhile, the HO-1 suppressor Zn (II) protoporphyrin IX (ZnPPIX) (Zn625, Frontier Scientific, Logan, UT, United States) was prepared based on a previous report (Francis et al., 2011). ZnPPIX was then diluted to 20μM in culture medium for 12 h, as described in a previous study (Kim et al., 2006). Nrf2 inhibitor ML385 (846557-71-9, Selleck Chemicals, Houston, TX, United States) was used at 15 μM in the medium for 24 h. The gentamicin + ML385 group received both gentamicin (0.6 mM) and ML385 (15 μM) for 24 h.

### RT^2^ Profiler Polymerase Chain Reaction Array

Approximately 20 organs of Corti were dissected from postnatal (P2) SD rats and cultured in medium with or without 0.6 mM gentamicin for 24 h. Total RNA extraction from the collected tissues was performed with the RNeasy Micro Kit (74004, QIAGEN, Hilden, Germany). The RT^2^ First Strand Kit (330401, QIAGEN, Hilden, Germany) was utilized for cDNA preparation from 1 μg of total RNA. The expression of 84 oxidative damage-related genes was assessed with the Oxidative Stress RT^2^ Profiler™ PCR Array kit (PARN-065ZC, QIAGEN, Hilden, Germany). The RT^2^ SYBR Green/ROX qPCR Mastermix (330522, QIAGEN, Hilden, Germany) was employed for quantitative real-time PCR (qPCR), as directed by the manufacturer, on an ABI ViiTM7 Real-Time PCR System in 25 μL reactions. The following protocol was used for amplification: 95°C, 10 min; 40 cycles of 95°C (15 s) and 60°C (60 s). The experiments were carried out in triplicate. The results of qPCR were uploaded on the RT^2^ Profiler™ PCR Array Data Analysis website.^2^ According to the instructions of the software, Ct cut-off value was set at 35. 2^–ΔCt^ was used to calculate the fold-change of mRNA expression. Student *t*-tests were used for the assessment of statistical significance. A *P*-value < 0.05 was considered statistically significant. The genes with *P* < 0.05 and more than 2-fold difference in expression were considered to be differentially expressed genes with biological significance.

### Gene Ontology Enrichment Analysis and Protein Interaction Network Construction

DAVID 6.8^3^ online analysis platform was used to annotate the screened differential genes in the GO and to classify processes or functions the differential genes mainly affect. R language was used to convert the data into a visual bubble chart. The protein interaction relationship of differentially expressed genes was analyzed through the STRING protein interaction database V11.0.^4^

### Quantitative Real-Time PCR

PCR was performed on the selected genes in order to verify the results of RT^2^ Profiler PCR Array. The entire organs of Corti were treated with 0 mM, 0.3 mM, 0.6 mM, and 1 mM gentamicin, after which they were collected. The total RNA was extracted with the RNeasy Micro Kit (74004, QIAGEN, Hilden, Germany). The RT^2^ First Strand Kit (330401, QIAGEN, Hilden, Germany) was utilized for cDNA preparation from 1 μg of total RNA. The following primers were employed: HO-1, sense 5′-TTTCACCTTCCCGAGCATC-3′ and antisense 5′-TCTTAGC CTCTTCTGTCACCCT-3′; β-actin, sense 5′-GAAGAGCTATG AGCTGCCTGA-3′ and antisense 5′-TGATCCACATCTGCTGG AAGG-3′; Nrf2, sense 5′-TTCCTCTGCTGCCATTAGTCAGTC-3′ and antisense 5′-GCTCTTCCATTTCCGAGTCACTG-3′; NQO1, sense 5′- GCGAGAAGAGCCCTGATTGTACTG -3′ and antisense 5′- TCTCAAACCAGCCTTTCAGAATGG -3′; GCLC, sense 5′- ACATCTACCACGCAGTCAAGGACC -3′ and antisense 5′- CTCAAGAACATCGCCTCCATTCAG -3′. β-actin was utilized for normalization. Data were obtained in triplicate and presented as mean.

### Cells Culture

Supporting cells were separated from the organs of Corti and cultured *in vitro*. The organs of Corti of three newborn rats (P2) were minced into small pieces and digested with PBS containing 0.5 mg/mL collagenase IV (17104-019, Gibco, Grand Island, NY, United States) for 20 min. DMEM/F-12 medium (11330032, Gibco, Grand Island, NY, United States) with 10% fetal bovine serum (FBS; 10091148, Gibco, Grand Island, NY, United States) and 1% antibiotic cocktail (penicillin and streptomycin; 15140-12, Gibco, Grand Island, NY, United States) were used to culture the cells in suspension for 24 h. Adherent-sphere cells were cultured in culture medium including factor DMEM/F-12, B-27™ Supplement (1:50, 17504044, Gibco, Grand Island, NY, United States), human EGF (5 ng/mL, AF100-15, PeproTech, Rocky Hill, NJ, United States), human FGF-basic (2.5 ng/mL, 100-18B, PeproTech, Rocky Hill, NJ, United States) and 1% antibiotic cocktail, in a humid environment containing 5% CO_2_ at 37°C for 24 h. Adherent cells were then transferred to DMEM/F-12 medium with 10% FBS and 1% antibiotic cocktail and cultured for additional 2 days.

HEI-OC-1 cells were cultured at 37°C with 5% CO_2_ in DMEM (C11995500BT, Gibco, Grand Island, NY, United States) containing 10% FBS (10099141, Gibco, Grand Island, NY, United States) and 1% penicillin (SV30010, HyClone, South Logan, UT, United States). The cells were subcultured at 80% confluence using 0.25% trypsin/EDTA (25200056, Gibco, Grand Island, NY, United States). When cells were cultured to a suitable density, the serum was removed, and cells were washed with PBS three times. CoPPIX (5 μM) and ZnPPIX (20 μM) were then added in the medium for 12 h.

### Immunofluorescence Staining

The entire organs of Corti underwent fixation with 4% paraformaldehyde for 8 h at 4°C. Supporting cells and HEI-OC1 cells underwent fixation with 4% paraformaldehyde for 20 min at room temperature. Blocking was carried out in phosphate-buffered saline (PBS) with 1% Triton X-100 (V900502, Sigma-Aldrich, St. Louis, MO, United States) and 5% bovine serum solution for 1 h. The tissue specimens underwent overnight incubation with rabbit anti-HO-1 (1:100, GTX101147, Gene Tex, Alton Pkwy Irvine, CA, United States), mouse anti-Nrf2 (1:200, ab89443, Abcam, Cambridge, MA, United States), goat anti-Sox2 (1:100, AF2018-SP, R&D Systems, Minneapolis, MN, United States), mouse anti-p27^Kip1^ (1:100, sc-1641, Santa Cruz, Santa Cruz, CA, United States), and rabbit anti-Myosin VII-a (1:800, 25-6790, Proteus Bioscience, Ramona, CA, United States) primary antibodies, respectively, at 4°C. Following four PBS washes, Alexa Fluor 488-linked donkey anti-mouse (AP192F, Millipore, Billerica, MA, United States), Alexa Fluor 594-linked donkey anti-rabbit (AP182C, Millipore, Billerica, MA, United States), and Alexa Fluor Plus 647 Donkey anti-Goat (H + L; A32849, Invitrogen, Carlsbad, CA, United States) secondary antibodies (1:200) were added to the specimens, respectively, for 12 h at 25°C. Rhodamine Phalloidin (1:200, PHDR1, Cytoskeleton, Denver, CO, United States) was used to mark HCs for 20min. Counterstaining was performed using 4′, 6′-diamidino-2-phenylindole (DAPI; 1:1000, D9542, Sigma-Aldrich, St. Louis, MO, United States). The entire specimen was evaluated in different turns under a confocal microscope (Nikon, Tokyo, Japan). 3D reconstructions were made by using Imaris (×64) software (Version: 9.0, Bitplane).

### Cell Counting

The cultured organs of Corti were placed under a confocal microscope. HCs of each turn were separately enumerated from micrographs acquired.

### MitoSOX Red Assay

Mitochondrial ROS amounts were assessed by MitoSOX Red staining (M36008, Invitrogen, Carlsbad, CA, United States). MitoSOX Red was used for the detection of mitochondrial reactive oxygen species by live-cell imaging. After organs of Corti were cultured with gentamicin (0.6 mM), CoPPIX (10 μM) or ZnPPIX (100 μM) for 48 h, the specimens underwent PBS washing and incubation with MitoSOX Red (5 μM) at 37°C in the presence of 5% CO_2_ for 10 min. Next, 4% paraformaldehyde was used for cell fixation at room temperature for 30 min before immunofluorescent staining.

### Western Blotting

The entire organs of Corti were treated in different groups. Total protein from cochlear specimens was obtained with the RIPA buffer that contained 1% PMSF. Nuclear–cytoplasmic fractionation was conducted using the NE-PER Nuclear and Cytoplasmic Extraction Reagents kit (Pierce, Thermo Fisher Scientific, Waltham, MA, United States) according to the manufacturer’s protocol. After clearing the lysate by centrifugation, the proteins were resolved by 8% SDS-PAGE and electro-transferred onto PVDF membranes. Upon blocking with 5% non-fat milk (1h at ambient), the membranes underwent overnight incubation with anti-HO-1 (1:100, GTX101147, Gene Tex, Alton Pkwy Irvine, CA, United States), anti-Nrf2 (1:100, ab89443, Abcam, Cambridge, MA, United States), and anti-GAPDH (1:1000, 10494-1-AP, Proteintech) and β-actin (1:1000, sc-47778, Santa Cruz, Santa Cruz, CA, United States) primary antibodies, respectively, at 4°C. This was followed by incubation with HRP-linked secondary antibodies (1:2000, SA00001-2, Proteintech) for 1h at ambient. The ECL kit (Pierce, Thermo Fisher Scientific, Waltham, MA, United States) was used for visualization.

### Statistical Analysis

SPSS 26 (SPSS Software, Chicago, IL, United States) and GraphPad 5.01 (GraphPad Software, San Diego, CA, United States) were used for data analysis. Data were compared by one-way analysis of variance (ANOVA). A *P*-value < 0.05 indicated statistically significant differences.

## Results

### Gentamicin Induced Gene Differential Expression

**FIGURE 1:**
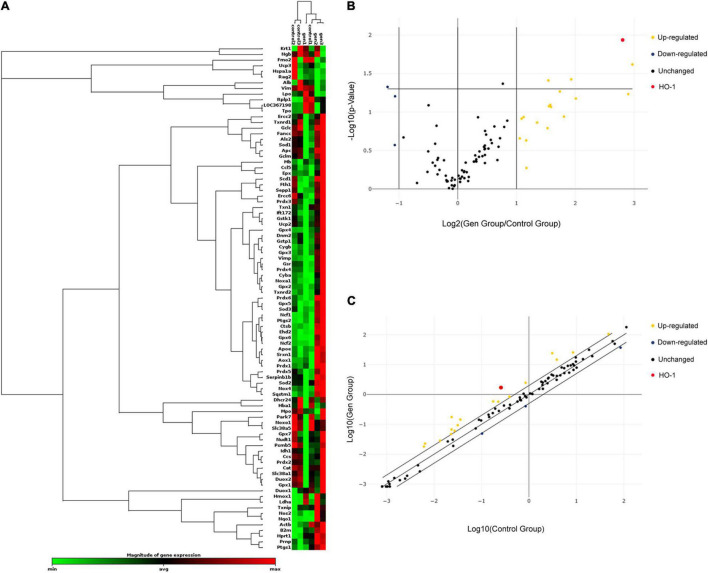
Relative expression of 84 oxidative stress-related genes in the control and gentamicin (0.6 mM) groups. **(A)** Hierarchical clustering of the 84 detected genes involved in oxidative stress in the cochlea, with expression levels ranging from low (green) to high (red). **(B)** The volcano plot shows notable gene expression changes between the control and gentamicin groups by plotting the log2 fold change in mRNA amounts on the x-axis and their statistical significance on the y-axis. **(C)** The scatter plot illustrates log-transformed relative expression levels for various genes (2^–ΔCt^) between the control group (x-axis) and the gentamicin group (y-axis).

The organs of Corti of the inner ear from 24 P2 rats were cultured in a medium with or without 0.6 mM gentamicin. After 24 h of culture, the RT^2^Profiler™ PCR Array Rat Oxidative Stress kit was used to investigate the expression of 84 known oxidative-related genes. Re-collect the organs of Corti and conduct three individual experiments. Gene expression assessment of triplicate assays was carried out as described on the RT^2^ Profiler™ PCR Array Data Analysis website (see text footnote 2), based on cycle threshold (Ct). Hierarchical clustering was used to evaluate the transcriptional levels of 84 oxidative stress-related genes in the cochlea (Figure 1A); volcano plots were used to compare gene expression between the control and gentamicin groups (Figure 1B). Figure 1C shows a scatter plot highlighting several notable genes based on large differences in expression fold between the control and gentamicin groups (Figure 1C). Of the 84 genes, 18 were up-regulated by more than 2-fold (Supplementary Table 1), and three genes were down-regulated by ≥2-fold (Supplementary Table 2). HO-1 was the gene with a relatively high up-regulation multiple which ranked the 3rd among these 21 genes, while with the smallest *p*-value in the whole group.

### Gene Ontology and Networks of the Differentially Expressed Genes

**FIGURE 2:**
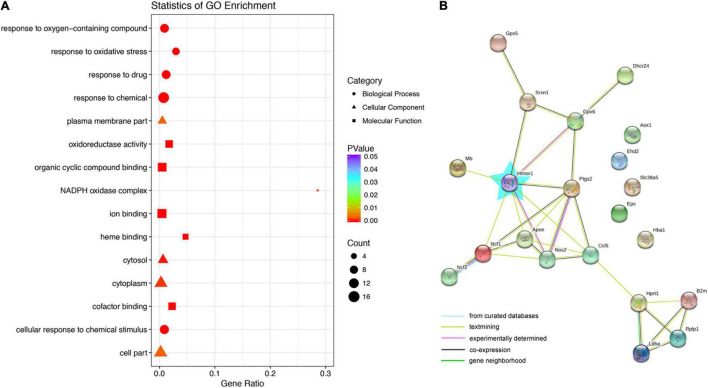
Gene ontology (GO) and network analysis of genes with differential expression between the control and gentamicin groups. **(A)** GO analysis of genes with differential expression between the control and gentamicin groups. **(B)** STRING protein-protein interaction analysis of genes. The blue star represents Hmox1 (HO-1).

To classify the processes and functions among genes with differential expression between the control and gentamicin groups, gene ontology (GO) analysis was carried out to categorize the 21 differentially expressed genes for their molecular functions (Figure 2A). Based on biological processes, these genes were involved in response to oxygen-containing compounds, cellular response to chemical stimulus, response to oxidative stress, response to the drug, and response to chemicals. Genes with altered expression in the gentamicin group were highly enriched in functional categories such as oxidoreductase activity, organic cyclic compound binding, ion binding, heme binding, and cofactor binding. The molecular components of the genes were highly enriched in the plasma membrane, cell part, NADPH oxidase complex, cytoplasm, and cytosol. In addition, a STRING protein-protein interaction network was constructed to visualize the possible connections between differentially expressed genes (Figure 2B). Among them, HO-1 showed an interaction with eight genes; PTGS2 interacted with six genes, and APOE and NOS2 interacted with five genes.

### Immunofluorescence Staining, QPCR, and Immunoblot Confirmed Heme Oxygenase-1 Expression

**FIGURE 3:**
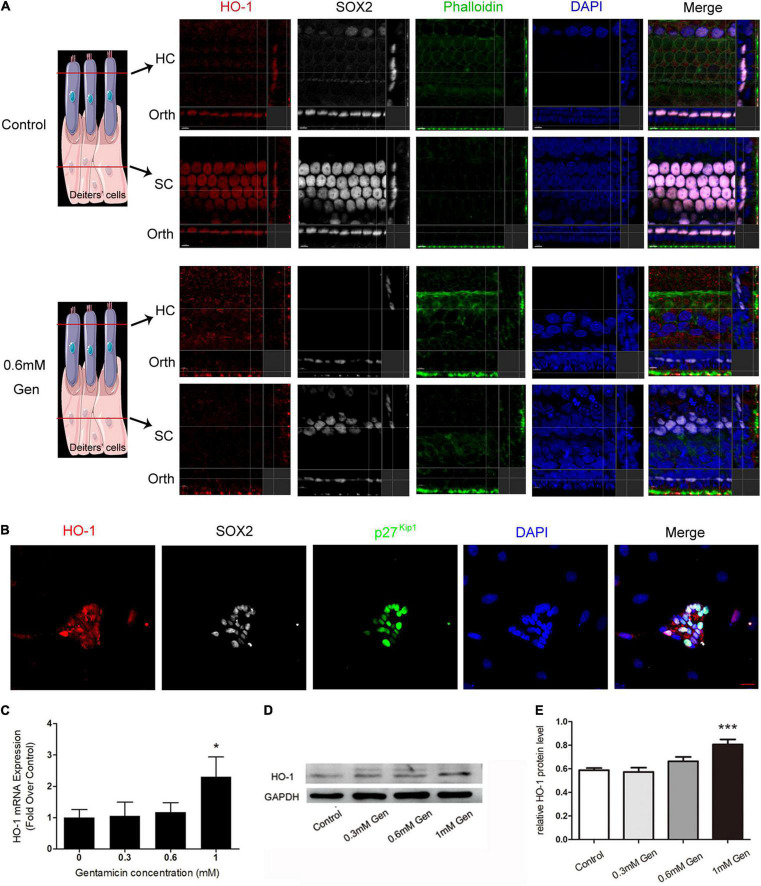
Verification of heme oxygenase-1 (HO-1) expression. **(A)** The localization of HO-1 (red), phalloidin (green), SOX2 (white), and DAPI (blue) before and after gentamicin (0.6 mM) treatment. The schematic showed the location of each panel. The upper panels show confocal images taken at the level of the hair cell cuticular plates; the lower panels show confocal images from the same field taken at the level of the supporting cell plates. Scale bar = 7 μm. Orth, orthogonal. **(B)** Micrographs depicting immunostaining for HO-1 in SCs without gentamicin *in vitro*. Scale bar = 20 μm. **(C)** The mRNA levels of HO-1 in the organs of Corti cultured separately in media containing different concentrations of gentamicin (*P < 0.05, *n* = 3). **(D)** Immunoblot detection of HO-1 in the organs of Corti at different gentamicin concentrations. **(E)** The signal of HO-1 (from D) quantitated after normalization to GAPDH and represented in bar diagrams (***P < 0.001, *n* = 3).

The organs of Corti were cultured in a medium with or without 0.6 mM gentamicin. After 24 h of culture, immunofluorescence staining showed that HO-1 highly expressed in SCs, and only slightly expressed in HCs in the control group. Treatment with 0.6 mM gentamicin increased the expression of HO-1 in HCs and decreased its expression in SCs (Figure 3A). SCs were separated from control group rats for *in vitro* culture. SOX2 and p27^Kip1^ are known markers of SCs. *In vitro* assays also confirmed that HO-1 was expressed in SCs in the control group (Figure 3B). In order to verify the expression of HO-1 up-regulated by gentamicin, the organs of Corti were treated with varying concentrations (0, 0.3, 0.6, and 1 mM) of gentamicin. The quantitative RT-PCR (qPCR) (Figure 3C) and immunoblot (Figures 3D,E) data indicated that gentamicin up-regulated HO-1 in a concentration-dependent manner (***P < 0.001, *P < 0.05, *n* = 3), which suggested that HO-1 participates in the process of gentamicin injury.

### Heme Oxygenase-1 Induction Inhibited Gentamicin-Related Hair Cell Death

**FIGURE 4:**
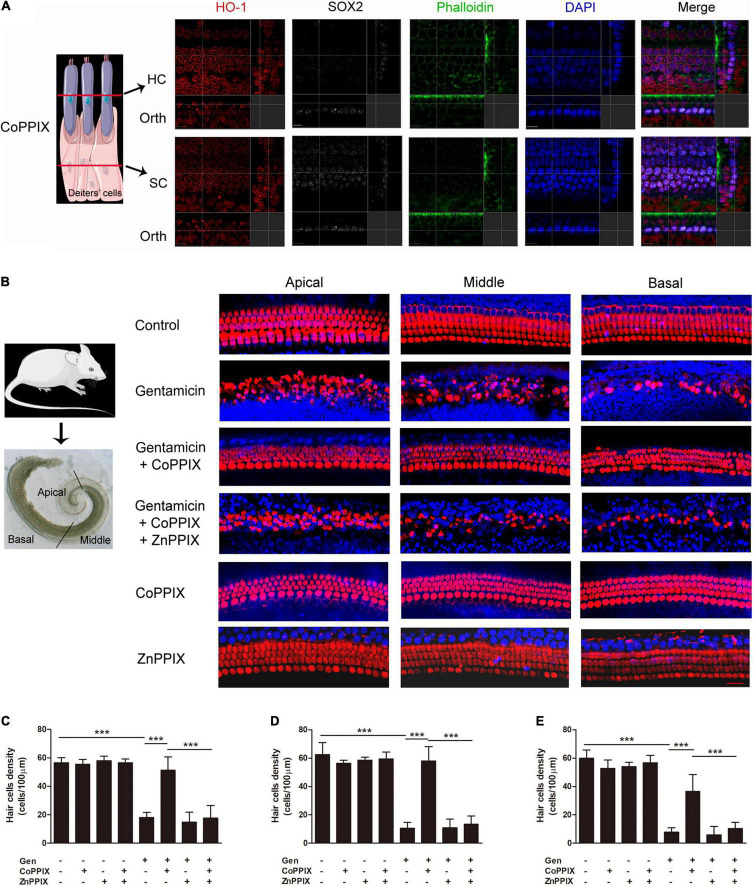
Heme oxygenase-1 (HO-1) activation inhibited gentamicin-associated hair cell death. **(A)** The localization of HO-1 (red), phalloidin (green), SOX2 (white), and DAPI (blue) after CoPPIX treatment. The schematic showed the location of each panel. The upper panels show confocal images taken at the level of the hair cell cuticular plates; the lower panels show confocal images from the same field taken at the level of the supporting cell plates. Scale bar = 7 μm. Orth, orthogonal. **(B)** Representative micrographs of immunofluorescent staining. Myosin VII-a was the marker of inner and outer HCs (red). Scale bar = 20 μm. **(C–E)** Amounts of surviving HCs in various groups upon gentamicin-induced damage in the apical, middle, and basal turns (***P < 0.001, *n* = 5).

To explore the role of HO-1 in the process of gentamicin injury, the HO-1 inducer CoPPIX (Ferrandiz and Devesa, 2008) was utilized to determine whether activating HO-1 protects HCs from gentamicin damage. The immunostaining showed that HO-1 induced in both HCs and SCs by CoPPIX (Figure 4A). The organs of Corti were pretreated with CoPPIX (5 μM) in the presence or absence of the HO-1 inhibitor ZnPPIX (20 μM) for 12 h and then further incubated with 0.6 mM gentamicin for 24 h. Myosin VII-a was used as a marker of inner and outer HCs. In control cells, the four HC rows were neatly arranged. Gentamicin administration for 24 h resulted in elevated HC loss in basal turns versus apical turns, forming a gradient from apex to base (Figure 4B); compared to the control group, the number of HCs in the apical turns was reduced by 68.20%, 83.01% in middle turns and approximately 87% in basal turn in the gentamicin group (***P < 0.001, *n* = 5) (Figures 4C–E, Formula for calculating ratio was (X-Y)/X. X means the number of HCs in each turn of control group, 56.6, 62.4, and 60, respectively. Y means the number of HCs in each turn of gentamicin group, 18, 10.6 and 7.8, respectively). Meanwhile, compared to the gentamicin group, the number of HCs in the apical, middle, and basal turns respectively increased by 185.56, 447.17, and 369.239% in the gentamicin + CoPPIX group (Figures 4C–E, Formula for calculating ratio was (Z-Y)/Z. Z means the average number of HCs in each turn of gentamicin + CoPPIX group, 51.4, 58, and 36.6, respectively). To further confirm that the protective effects involved HO-1 activation, the HO-1 inhibitor ZnPPIX (Wong et al., 2011) was applied. The gentamicin + CoPPIX + ZnPPIX group showed no protection by CoPPIX in the apical, middle, and basal turns (Figures 4B–E). We confirmed that HO-1 was required for the protection against gentamicin-induced HC death. No loss of HCs was observed in the CoPPIX and ZnPPIX groups. Furthermore, HC counts showed the same trend in cell density changes from apical to basal turns in various groups (Figures 4C–E).

### Heme Oxygenase-1 Induction Decreased Intracellular Reactive Oxygen Species Levels

**FIGURE 5:**
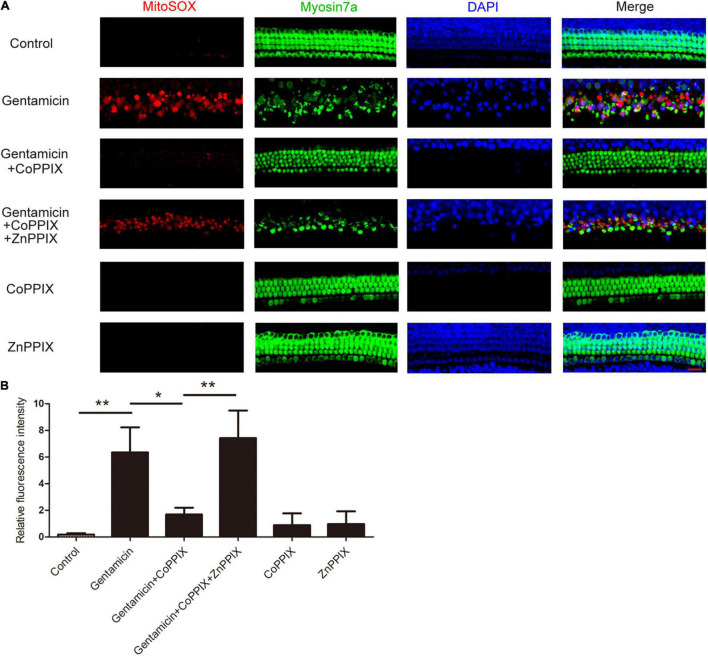
Induction of heme oxygenase-1 (HO-1) by CoPPIX reduces intracellular reactive oxygen species (ROS) levels, and ZnPPIX increases intracellular ROS levels. **(A)** Representative micrographs showing MitoSOX Red immunostaining. The organs of Corti were pretreated with CoPPIX (5 μM) in the presence or absence of ZnPPIX (20 μM) for 12 h and then further incubated with 0.6mM gentamicin for 24 h. Scale bar = 20 μm. **(B)** Quantification of relative fluorescence intensities of ROS in each group (*P < 0.05 and **P < 0.01, *n* = 3).

MitoSOX Red, a redox fluorophore specifically measuring superoxide amounts in the mitochondria, was utilized to investigate the effects of HO-1 on ROS production after gentamicin treatment. Immunofluorescence staining revealed that the amount of ROS in cochlear HCs treated with gentamicin increased to 34.73-times of that in the control group. In the gentamicin +CoPPIX group, the amounts of ROS-positive HCs decreased by 73.55% compared to gentamicin group. The gentamicin + CoPPIX + ZnPPIX group exhibited more ROS-positive HCs than the gentamicin + CoPPIX group. ROS were seldom found in the control, CoPPIX and ZnPPIX groups (**P < 0.01 and *P < 0.05, *n* = 3) (Figures 5A,B).

### Gentamicin Induced Heme Oxygenase-1 Activation via Nrf2 Signaling

**FIGURE 6:**
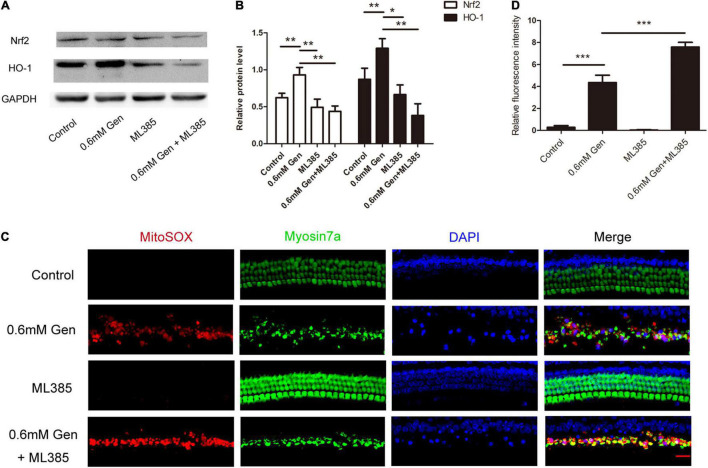
Gentamicin induced heme oxygenase-1 (HO-1) activation via Nrf2 signaling. **(A)** The level of Nrf2 and HO-1 expression was determined by Western blot in different groups at 24 h after culture. **(B)** The signal of Nrf2 and HO-1 [from **(A)**] quantitated after normalization to β- actin and represented in bar diagrams (*P < 0.05, **P < 0.01, ***P < 0.001, *n* = 4). **(C)** Representative micrographs showing MitoSOX Red immunostaining in different groups. **(D)** Quantification of relative fluorescence intensities of reactive oxygen species (ROS) in each group (***P < 0.001, *n* = 3).

To further investigate the pathway, through which gentamicin up-regulated HO-1, Nrf2 inhibitor ML385 was used. In the experiment, the organs of Corti were randomly divided into four groups: the control group, the gentamicin group, the ML385 group only received ML385 (15 μM), and the gentamicin + ML385 group. Immunoblot showed a significant increase in Nrf2 and HO-1 levels after 0.6 mM gentamicin. Nrf2 and HO-1 levels were not suppressed significantly in the organs of Corti following treatment with Nrf2 inhibitors ML385. However, compared with the gentamicin group, ML385 inhibited both Nrf2 and HO-1 induced by gentamicin (***P < 0.001 and *P < 0.05, *n* = 4) (Figures 6A,B). Immunofluorescence staining revealed that the ML385 + gentamicin group exhibited more ROS-positive HCs than the gentamicin group. ROS were seldom found in the control and ML385 groups (***P < 0.001, *n* = 3) (Figures 6C,D). These suggested that disrupting Nrf2 activity prevents the upregulation of HO-1 and the protection after gentamicin treatment.

### Heme Oxygenase-1 Induction Increased the Expression of Nrf2

**FIGURE 7:**
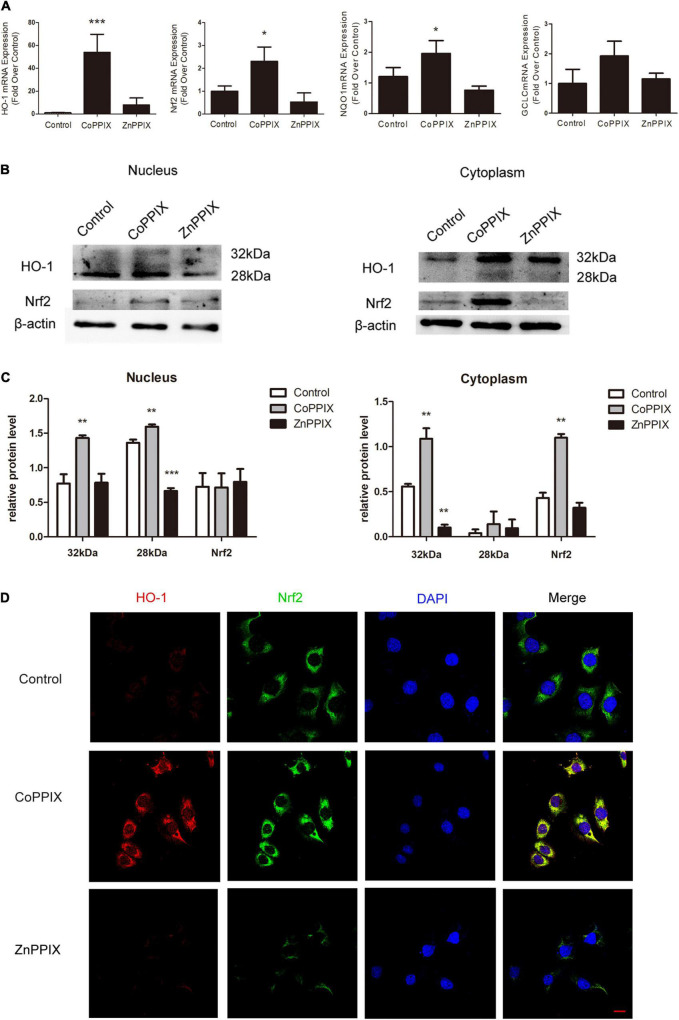
Heme oxygenase-1 (HO-1) induction increased the expression of Nrf2. **(A)** The mRNA levels of HO-1, Nrf2, NQO1, and GCLC in the organs of Corti incubated with CoPPIX or ZnPPIX for 12 h (*P < 0.05, ***P < 0.001, *n* = 3). **(B)** The level of HO-1 and Nrf2 expression were determined by Western blot in the nuclear and cytoplasmic fractions of the organs of Corti incubated with CoPPIX or ZnPPIX for 12 h. **(C)** The signal of HO-1 and Nrf2 [from **(B)**] quantitated after normalization to β- actin and represented in bar diagrams (*P < 0.05, **P < 0.01, n = 3). **(D)** Immunofluorescence staining of HEI-OC1 in different groups. Scale bar = 20 μm.

Nrf2 transcriptionally activates several antioxidant genes, including NAD(P)H: quinone oxidoreductase 1 (NQO1) and gamma- glutamate cysteine ligase catalytic subunit (GCLC). mRNA levels of HO-1, Nrf2, and Nrf2 target genes were analyzed by qPCR. CoPPIX treatment of the organs of Corti remarkably up-regulated HO-1 mRNA expression. Further, mRNA levels of Nrf2, NQO1 and GCLC increased in response to CoPPIX treatment (***P < 0.001, *P < 0.05, *n* = 3) (Figure 7A). CoPPIX and ZnPPIX treatment of the organs of Corti for 12 h were analyzed for HO-1 and Nrf2 proteins by Western blot. In the nucleus, two HO-1 immunoreactive bands were observed upon Western analysis, with one band migrating at 28 kDa and the other migrating at 32 kDa. In the nucleus CoPPIX treatment of the organs of Corti, 32 kDa HO-1 and 28 kDa HO-1 were up-regulated at the same time. ZnPPIX treatment inhibited 28 kDa HO-1 but not 32 kDa HO-1 compared with the control group. There were no significant changes in nuclear Nrf2 whether CoPPIX or ZnPPIX treatment. After CoPPIX treatment, HO-1 in the cytoplasm appeared a band at 32 kDa and a faint band at 28 kDa, which was accompanied with a simultaneous increase of Nrf2. ZnPPIX treatment inhibited 32 kDa HO-1 in the cytoplasm significantly. However, neither 28 kDa HO-1 nor Nrf2 has changed significantly after ZnPPIX treatment (*P < 0.05, **P < 0.01, *n* = 3) (Figures 7B,C). Immunofluorescence staining of HEI-OC1 cells verified that the expression of Nrf2 was induced by the HO-1 induction. In addition, Nrf2 was mainly accumulated in the cytoplasm (Figure 7D). These data suggested that HO-1 may modulate the accumulation of Nrf2.

## Discussion

Reactive oxygen species formation is one of the key mediators of aminoglycoside-induced HC death (He et al., 2017, 2020). Gentamicin generates free radicals within the inner ear, including the highly reactive hydroxyl radical and lipid peroxidation products (Sha and Schacht, 1999). Previous reports have also revealed that antioxidants may prevent gentamicin-induced cochlear damage involving ROS (Niwa et al., 2016). The present study assessed the expression of oxidative damage-related genes as well as the mechanism of drug ototoxicity. We examined oxidative damage-related genes after gentamicin treatment for 24 h, the late stage of redox reactions. Our results revealed that HO-1 levels on the organs of Corti after gentamicin treatment increased 6.99 times (*p* < 0.05) compared to control values. In addition, we also found that HO-1 interacted with the other eight genes (Mb, Ncf1, Apoe, Nos2, Pstgs2, Gpx6, Srxn1, Ccl5). Based on this, we speculated that HO-1 played a role in gentamicin injury.

Our study showed that HO-1 slightly expressed in the inner and outer HCs and SCs in normal rat cochlea. After gentamicin treatment, HO-1 significantly decreased in SCs, while it increased in the inner and outer HCs. Compared with the control group, CoPPIX induced an increase in the expression of HO-1 mainly in HCs. Fairfield et al. (2004) found a constitutive but limited production of HO-1 in the outer HCs of normal rat cochlea. Furthermore, the expression of HO-1 significantly increased after hyperthermia, being selectively localized in all three rows of outer HCs in the organ of Corti as well as in marginal and intermediate cells of stria vascularis (Fairfield et al., 2004). Previous studies have reported on supporting cells as critical determinants of whether a hair cell under stress ultimately lives or dies (May et al., 2013). Yet, the mechanisms through which hair cells send stress signals and supporting cells sense and respond to these signals remain unclear.

In this study, we found that the induction of HO-1 by CoPPIX inhibited ROS production and reduced the damage of gentamicin to HCs. These findings suggested that HO-1 induction by CoPPIX might contribute to HC protection against gentamicin-induced damage. Recent evidence also suggested that HO-1 was essential in modulating antioxidant effects in other tissues (Shalaby et al., 2019; Niu et al., 2020). Park et al. (2017) found that in the cochlear tissue, a peroxisome proliferator-activated receptor (PPAR) inducer protected HCs from gentamicin-induced toxicity by increasing the expression of HO-1. Based on previous studies, herein, we focused on the location of the up-regulated expression of HO-1 after gentamicin and the signal pathway of HO-1. Therefore, specifically activating HO-1 gene expression by pharmacological regulation might constitute a new treatment target for gentamicin-related cochlear ototoxicity.

Our results revealed that while HO-1 was regulated by Nrf2, it could also promote the accumulation of Nrf2. HO-1 can prevent the damage of gentamicin by mutually regulating Nrf2. Nrf2/HO-1 is a classic signaling pathway in antioxidant reactions. Under oxidative stress conditions, the transcription factor Nrf2 undergoes nuclear translocation and regulates the expression of corresponding downstream genes, thus reducing oxidative stress (Fetoni et al., 2015; Ni et al., 2017). Up-regulation of Nrf2/HO-1 signaling alleviates gentamicin-induced nephrotoxicity (Subramanian et al., 2014; He et al., 2015). Celastrol activates the Nrf2-transcription factor and induces HO-1, which inhibits pro-apoptotic JNK activation and HC death, possibly through the action of CO (Francis et al., 2011). Although it is well known that Nrf2 induces HO-1 leading to mitigation of oxidant stress, evidence proves that nuclear isoform of HO-1 could also regulate Nrf2 activation by using hyperoxia exposure in mouse embryonic fibroblasts (MEFs) (Biswas et al., 2014). Our study found that there were two HO-1 immunoreactive bands in whole cell lysates upon Western analysis. Same as 32 kDa HO-1, the expression of 28 kDa HO-1 increases with the increase of gentamicin dose. Our study suggested that the 32 kDa isoform constitutively was predominant in the cytoplasm, whereas, the 28 kDa HO-1 was primarily localized to the nucleus. Different from the results of Biswas, we found that the Nrf2 up-regulated by 32 kDa HO-1 in the cytoplasm, not in the nucleus. Participation of various signaling pathways has been implicated in Nrf2 transcription. Accumulation and nucleocytoplasmic trafficking of Nrf2 may be affected by phosphorylation. GSK3β, a kinase that sensitizes cells for cell death, can phosphorylate and activate Fyn kinase, leading to phosphorylation, nuclear exclusion and accumulation in the cytoplasm of Nrf2 protein (Salazar et al., 2006). In addition, CoPPIX is a potent inducer of HO-1. Many data indicated that CoPPIX had a significant induction of HO-1 and the induction of HO-1 played a protective role in other system diseases (Chora et al., 2007; Song et al., 2018). However, it is not clear whether COPPIX only upregulates HO-1, but not other proteins, which causes the upregulation of Nrf2. The fact that HO-1 regulates the accumulation of Nrf2 in the cytoplasm still needs further verification.

## Conclusion

Gentamicin-associated HC death represents a complex process involving the changes in protein expression of different cell types as well as the signaling pathways in HCs. In this study, we found that HO-1 protects HCs from gentamicin by up-regulating its expression in HCs and interacting with Nrf2 to inhibit ROS. Our results also suggest that HO-1 could be considered as a candidate target for designing regimens to efficiently prevent gentamicin-associated hearing loss. In the next step, we plan to use new technologies such as hydrogels and nanoparticles to deliver CoPPIX to the inner ear so as to study the protective effect of HO-1 on gentamicin-induced hearing loss *in vivo*.

## Data Availability Statement

The original contributions presented in the study are included in the article/Supplementary Material, further inquiries can be directed to the corresponding author/s.

## Ethics Statement

The animal study was reviewed and approved by Air Force Military Medical University.

## Author Contributions

YY: conceptualization, investigation, methodology, validation, data curation, and writing—original draft. XC: methodology and writing—original draft. KYT: methodology and software. CYT: methodology and picture processing. LYC: data curation. WJM: picture processing and software. QL: writing—reviewing and editing. JHQ: conceptualization and writing—reviewing and editing. YL: methodology, project administration, and funding acquisition. DJZ: conceptualization, writing—reviewing and editing, and funding acquisition. All authors contributed to the article and approved the submitted version.

## Conflict of Interest

LC was employed by Smartgenomics Technology Institute. The remaining authors declare that the research was conducted in the absence of any commercial or financial relationships that could be construed as a potential conflict of interest.

## Publisher’s Note

All claims expressed in this article are solely those of the authors and do not necessarily represent those of their affiliated organizations, or those of the publisher, the editors and the reviewers. Any product that may be evaluated in this article, or claim that may be made by its manufacturer, is not guaranteed or endorsed by the publisher.

## Footnotes

1
https://www.who.int/health-topics/hearing-loss#tab=tab_1

2
https://geneglobe.qiagen.com/cn/analyze/

3
https://david.ncifcrf.gov/

4
https://string-db.org/

## Abbreviations

HC: hair cell
SC: supporting cell
HO-1: heme oxygenase-1
CoPPIX: Co (III) protoporphyrin IX chloride
ZnPPIX: Zn (II) protoporphyrin IX
Nrf2: nuclear factor erythroid 2 (NF-E2)-related factor 2
ROS: reactive oxygen species
P2: postnatal day 2
SD: sprague-dawley
PBS: phosphate buffered saline
DAPI, 4′: 6′-diamidino-2-phenylindole
GAPDH: glyceraldehyde-3-phosphate dehydrogenase
GO: gene ontology
NADPH: nicotinamide adenine dinucleotide phosphate
NMDA: *N*-methyl -*D*-aspartate
PCR: polymerase chain reaction
qPCR: quantitative real-time polymerase chain reaction.
