# Supplementary material for: Heme Oxygenase-1 Protects Hair Cells From Gentamicin-Induced Death
Source: Front Cell Neurosci. 2022 Apr 13;16:783346. doi: 10.3389/fncel.2022.783346 (PMC9043494; doi:10.3389/fncel.2022.783346)
Supplement: Supplementary file 1 [file Table_1.DOCX]

| **Position** | **RefSeq Number** | **Gene title** | | **Gene**  **symbol** | **Fold**  **change** | **P-value** |
| --- | --- | --- | --- | --- | --- | --- |
| H04 | NM_017025 | Lactate dehydrogenase A | | Ldha | 7.84 | 0.024044 |
| D09 | NM_053734 | Neutrophil cytosolic factor 1 | | Ncf1 | 7.47 | 0.058416 |
| C12 | NM_012580 | Heme oxygenase (decycling) 1 | | Hmox1 | 6.99 | 0.011630 |
| F04 | NM_017232 | | Prostaglandin-endoperoxide synthase 2 | Ptgs2 | 4.02 | 0.066832 |
| H02 | NM_012512 | | Beta-2 microglobulin | B2m | 3.81 | 0.037626 |
| D10 | NM_001100984 | | Neutrophil cytosolic factor 2 | Ncf2 | 3.50 | 0.114642 |
| A03 | NM_019363 | | Aldehyde oxidase 1 | Aox1 | 3.33 | 0.054060 |
| A05 | NM_138828 | | Apolipoprotein E | Apoe | 3.01 | 0.085762 |
| G04 | NM_001047858 | | Sulfiredoxin 1 homolog | Srxn1 | 2.98 | 0.080706 |
| D07 | NM_021588 | | Myoglobin | Mb | 2.93 | 0.084083 |
| H03 | NM_012583 | | Hypoxanthine phosphoribosyltransferase 1 | Hprt1 | 2.91 | 0.038816 |
| C05 | NM_001105738 | | Glutathione peroxidase 5 | Gpx5 | 2.88 | 0.162027 |
| C06 | NM_147165 | | Glutathione peroxidase 6 | Gpx6 | 2.55 | 0.136870 |
| H05 | NM_001007604 | | Ribosomal protein, large, P1 | Rplp1 | 2.25 | 0.533085 |
| A07 | NM_031116 | | Chemokine (C-C motif) ligand 5 | Ccl5 | 2.24 | 0.233748 |
| B04 | NM_001024897 | | EH-domain containing 2 | Ehd2 | 2.17 | 0.116289 |
| B05 | NM_001107037 | | Eosinophil peroxidase | Epx | 2.12 | 0.121972 |
| D12 | NM_012611 | | Nitric oxide synthase 2, | Nos2 | 2.08 | 0.219923 |

**Table 1**

List of selected genes increased by 2-fold or more.

**Position**, the number assigned to each gene by PCR Array.
