# Supplementary material for: Heme Oxygenase-1 Protects Hair Cells From Gentamicin-Induced Death
Source: Front Cell Neurosci. 2022 Apr 13;16:783346. doi: 10.3389/fncel.2022.783346 (PMC9043494; doi:10.3389/fncel.2022.783346)
Supplement: Supplementary file 2 [file Table_2.DOCX]

**Table 2**

List of selected genes decreased to 0.5-fold or less.

| **Position** | **RefSeq Number** | **Gene title** | **Gene**  **symbol** | **Fold**  **change** | **P-value** |
| --- | --- | --- | --- | --- | --- |
| C11 | NM_013096 | Hemoglobin alpha, adult chain 2 | Hba1 | -2.29 | 0.047099 |
| A12 | NM_001080148 | 24-dehydrocholesterol reductase | Dhcr24 | -2.10 | 0.268647 |
| F11 | NM_138854 | Solute carrier family 38, member 5 | Slc38a5 | -2.09 | 0.062365 |

**Position**, the number assigned to each gene by PCR Array.
